# Could choosing risdiplam instead of nusinersen in the treatment of type 1 spinal muscular atrophy be a huge cost-minimization opportunity?

**DOI:** 10.3325/cmj.2024.65.454

**Published:** 2024-10

**Authors:** Andrej Belančić, Andrea Katrin Faour, Elvira Meni Maria Gkrinia, Dinko Vitezić

**Affiliations:** 1 Department of Clinical Pharmacology, Clinical Hospital Centre Rijeka, Rijeka, Croatia *a.belancic93@gmail.com*; 2 Department of Basic and Clinical Pharmacology with Toxicology, University of Rijeka, Faculty of Medicine, Rijeka, Croatia; 3 Vancouver Coastal Health, Vancouver, BC, Canada; 4 Independent Researcher, Athens, Greece

Until 2016, spinal muscular atrophy (SMA) was the most common inherited cause of infant death (1). A huge paradigm shift occurred with the development of three orphan medicines (intrathecal antisense oligonucleotide – nusinersen, intravenous gene replacement therapy – onasemnogene abeparvovec-xioi, and peroral small-molecule splicing modifier – risdiplam), which are compensating for the deficient SMN protein, extending lifespan, and improving the quality of life in infants and children with SMA. The efficacy and safety of these medicines in infantile-onset SMA were evaluated in the ENDEAR, STR1VE, and FIREFISH trials, respectively (2-4).

An issue worth highlighting is the small number of head-to-head comparisons between orphan medicines in general, with historical controls/best supportive care being the most common comparator. Comparing orphan medicines with best supportive care limits decision-making and designing of health policy around rare diseases.

Ribero et al reported the results of a well-designed systematic literature review and indirect treatment comparison (5). Although no concrete conclusions were drawn from the indirect comparison analyses between onasemnogene abeparvovec-xioi and nusinersen, risdiplam was associated with improved outcomes over nusinersen in SMA type 1 (5). In particular, matching-adjusted indirect comparison analyses vs ENDEAR demonstrated that, compared with nusinersen, risdiplam may extend event-free survival and overall survival, as well as improve achievement of some motor milestones in infants with SMA type 1. Also, despite the longer follow-up period, risdiplam was associated with a lower likelihood of reporting serious adverse events (5). After the nusinersen-to-risdiplam switch was evaluated as safe (JEWELFISH study and Kwon et al expanded access program report) (6,7), in the absence of direct head-to-head comparisons, we have conducted a pioneer real-world study assessing the effectiveness and safety of these medicines in the “Croatian Nusinersen-Risdiplam Switch Cohort” (8). Our results demonstrated risdiplam’s non-inferiority in the SMA type 1 setting (eg, +1.0 in CHOP INTEND score; *P* = 0.067) (8). These findings are important as patients with SMA (type 1) frequently need to switch treatments due to personal or clinical reasons, such as inadequate clinical response, convenience of administration (especially in cases of scoliosis or scoliosis surgery-related spinal fusion), and/or medicine- or administration-related adverse events. Here, we have identified a few opportunities linked with risdiplam use in the SMA type 1 setting that we would like to present as an adjunct to the recent reports by Yeo et al and Sansone (1,9). Thus, under the clinical equivalence hypothesis, we aimed to perform a European budget impact analysis comparing the costs of risdiplam and nusinersen in the management of SMA type 1, which is the most prevalent and most severe clinical phenotype of SMA. Hopefully, this commentary will potentially bring down the prices of orphan medicines for the treatment of SMA, lead to money savings and better translocation of financial resources within the health care system, and improve the quality of life and care of patients living with SMA type 1.

According to the Agency for Medicinal Products and Medical Devices of Croatia (as the best available pricing data obtained through literature searches in February 2024), the highest permitted wholesale prices of risdiplam (Evrysdi®) 0.75-mg/mL bottle (containing 60 mg of risdiplam in 2-g powder for oral solution) and nusinersen (Spinraza®) 5-mL vial (containing 12 mg of nusinersen) for 2023 were €5,679.63 and €70,353.83, respectively (10). Over a one-year (eg, 366 days) period, a single patient with SMA type 1 (taking the maximum administration doses – 5 mg/day for risdiplam and 12 mg/application for nusinersen) would need 30.5 bottles and 6 vials, respectively. If we apply the highest permitted wholesale prices and the maximum administration doses of the medicines, by choosing risdiplam over nusinersen in the SMA type 1 setting, one could save €248,894.265 per patient [(6 × €70,353.83) – (30.5 × €5,679.63)] in one year. Considering differences in the dosing regimen of nusinersen in the following years (3 vials/y), one would annually save up to €37,832.775 [(3 × €70,353.83) – (30.5 × €5,679.63)] for each upcoming year; thus, the overall savings with risdiplam, over a five-year period, would be €400,225.365 per patient [€248,894.265 + (4 × €37,832.775)].

According to Verhart et al, 4653 patients were genetically diagnosed with SMA in Europe in a five-year period (2011-2015) (11). Today, this number might be even higher due to wider implementation of new-born screening into practice. Under the assumption that the prevalence of SMA type 1 is nearly 60% (N = 2790), and that the extrapolated number of annual live births in a five-year period is fixed (N = 558), one would save €138,883,000.00 (558 × €248,894.265) in the first year, and later annually as follows: [(558 × €248,894.265) + (558 × €37,832.775)], [(558 × €248,894.265) + (1116 × €37,832.775)], [(558 × €248,894.265) + (1674 × €37,832.775)], and [(558 × €248,894.265) + (2232 × €37,832.775)].

Under our hypothesis, we here present how Europe can potentially save up to €905,521,883.9 [(5 × 558 × €248,894.265) + (10 × 558 × €37,832.775)] in the next five years if nusinersen is switched to risdiplam in newly diagnosed patients with SMA type 1. The cost-savings of such a strategy may increase further with the implementation of new-born screening and early risdiplam initiation. Apart from this, other potential advantages to the shift in prescription can be found in the safety profile and the patient/caregiver perspective (12,13). Powell et al have recently reported that most patients were satisfied when switching from nusinersen to risdiplam, with the route of administration being the most important factor (13).

This is a cost-minimization analysis, therefore, the findings should be interpreted accordingly (bearing in mind the orphan field and the availability of the current body of literature) (14,15). Given the rarity of the disease, data from formal studies and real-world settings are likely obtainable from registries or company sources. Thus, these data should be made accessible to enable the use of matching or weighting techniques, which could emulate randomized scenarios and accurately compare the effectiveness and safety relationship between the disease-modifying drugs. Another issue is that the costs of neither risdiplam nor nusinersen are fixed, thus market forces could lead to their prices potentially being modified; however, we recognize that this is merely a hypothesis. One may point out the differences in the national wholesale prices of risdiplam and nusinersen as a limitation of the European budget impact calculations. However, the savings linked with risdiplam use in the setting of SMA type 1 could be even more substantial than presented, since there are no costs related to intrathecal administration or post-lumbar puncture side-effects management, and the dose of risdiplam in patients younger than 2 years of age and weighing less than 20 kg is less than 5 mg per day (ranging from 0.15 to 0.25 mg/kg).

To conclude, pharmacoeconomic projections such as this, based on longer-term prospective outcome (re)evaluations for orphan medicines, may modify drug administration criteria and reimbursement guidelines of the national health insurance fund, and consequently lead to significant savings and a shift in the accompanied financial resources to other indications and health care strategies.
